# ASPP2 inhibits hepatitis B virus replication by preventing nucleus translocation of HSF1 and attenuating the transactivation of ATG7

**DOI:** 10.1111/jcmm.16699

**Published:** 2021-06-04

**Authors:** Shanshan Wang, Yu Sun, Yang Wang, Anna Wang, Buxin Kou, Yang Che, Dexi Chen, Yulin Zhang, Ying Shi

**Affiliations:** ^1^ Capital Medical University Affiliated Beijing You’an Hospital Beijing China; ^2^ Beijing Institute of Hepatology Beijing China; ^3^ Minimally Invasive Interventional Centre of Oncology Beijing You’an Hospital Capital Medical University Beijing China

**Keywords:** ASPP2, Atg7, autophagy, HBV, HSF1

## Abstract

Hepatitis B virus (HBV) is a kind of virus with the capability to induce autophagy, thereby facilitating its replication. Reducing hepatocyte autophagy is proved to be a useful way to inhibit HBV replication. Herein, we reported that p53‐binding protein 2 (apoptosis‐stimulating protein of p53‐2, ASPP2) could attenuate HBV‐induced hepatocyte autophagy in a p53‐independent manner. Mechanistically, overexpressed ASPP2 binds to HSF1 in cytoplasm of HBV‐infected cells, which prevents the translocation of HSF1 to nuclei, thereby inhibiting the transactivation of Atg7. By regulating the transcription of Atg7, ASPP2 reduces hepatocyte autophagy, thereby inhibiting HBV replication. Therefore, ASPP2 is a key regulator of cell autophagy, and overexpression of ASPP2 could be a novel method to inhibit HBV replication in hepatocytes.

## 1 INTRODUCTION

Hepatitis B virus (HBV) infection is a global public health problem, which can result in chronic hepatitis, hepatic cirrhosis and even hepatocellular carcinoma (HCC). It is estimated that 240 million people are chronically infected with HBV all over the world,^1^ and HBV infection contributes to half mortality of HCC in 2010.^2^ So, complete elimination of HBV from human hepatocytes is very urgent. Autophagy is known as a catabolic process that is beneficial for the maintenance of cellular homeostasis. In addition, autophagy can act as a cellular defence against virus infection and promote virus clearance. Conversely, invaded virus strives to avoid the attack from cellular autophagy and destroy the intracellular homeostasis.^3^, ^4^ Interestingly, HBV can induce autophagy and even promote self‐replication through autophagy.^5^, ^6^, ^7^, ^8^, ^9^ Currently, many in vitro and in vivo studies have demonstrated that the induction or suppression of autophagy can promote or inhibit HBV replication.^10^, ^11^, ^12^, ^13^ Therefore, autophagy is probably a new target to inhibit HBV replication.

ASPP2 is a member of p53‐binding protein family, which is initially found to bind p53 and promote p53‐mediated apoptosis. Recent studies have also found that ASPP2 participates in a variety of cellular processes in a p53‐independent manner.^14^ ASPP2 is considered to be a tumour suppressor. The down‐regulation of ASPP2 is observed in the majority of human cancers, including hepatocellular carcinoma,^15^ pancreatic cancer^16^ and squamous cell carcinoma.^17^ Recent studies have confirmed that ASPP2 is involved in the regulation of autophagy by interacting with different molecules. The deficiency of ASPP2 can inhibit autophagy, thus ameliorating acute kidney injury induced by ischaemia reperfusion.^18^ Similarly, ASPP2 can suppress methionine and choline‐deficient (MCD) diet‐induced autophagy and reduce liver injury in a mouse model.^19^ ASPP2 also can inhibit RAS‐induced autophagy by preventing the formation of ATG16‐ATG5‐ATG12 complex.^20^ In this study, whether ASPP2 can inhibit HBV replication through negatively regulating hepatocellular autophagy and its possible mechanisms are explored.

## 2 MATERIALS AND METHODS

### 2.1 Cell culture, plasmids and small interfering RNA

Cell lines (HepG2, Huh7, HepG2.2.15 (HBV positive) and Hep3B (p53 null)) were grown in Dulbecco's modified Eagle's medium (DMEM) with 10% foetal bovine serum, 290 μg L‐glutamine, 100 units penicillin and 100 μg streptomycin (Invitrogen Life Technology) per 500 mL under the environment of 37°C and 5% CO_2_.

HBV 1.28mer plasmid was a gift kindly provided by Dr James Ou (University of South California). His‐ASPP2, His‐control and pcDNA4‐HSF1 plasmids were obtained from Beijing Biomed Biology Co. Ltd. The primer sequences of siRNA against ASPP2, Atg7 and scrambled siCon (GenePharma, A10001) were listed as follows (Table 1).

**TABLE 1:** siRNA used in this work

Target RNA	siRNA forward	siRNA reverse
Atg7#1	GGUCAAAGGACGAAGAUAAdTdT	UUAUCUUCGUCCUUUGACCdTdT
Atg7#2	GCAUCAUCUUCGAAGUGAAdTdT	UUCACUUCGAAGAUGAUGCdTdT
Atg7#3	CAAGGUUGUGUCUGUCAAAdTdT	UUUGACAGACACAACCUUGdTdT
ASPP2#1	GCCCAAAUCUCCAGAAUAATT	UUAUUCUGGAGAUUUGGGCTT
ASPP2#2	GAGACUAAGCAAUGGGAAATT	UUUCCCAUUGCUUAGUCUCTT
ASPP2#3	GGACUGUACCCAAGAAUUATT	UAAUUCUUGGGUACAGUCCTT

HBsAg and HBeAg were detected by enzyme‐linked immunosorbent assay (ELISA).

In order to detect HBsAg, the supernatant of cell culture was examined by ELISA according to the manufacturer's instructions (Zhongshan Bio‐Tech, Guangdong, China). Each experiment was performed at least three times.

### 2.2 HBV DNA detection

The supernatant of cell culture was collected to determine HBV DNA by using Hepatitis B virus Quantitative Detection Kit (Sansure Biotech, Hunan, China) according to the manufacturer's instructions.

### 2.3 Western blot analysis and co‐immunoprecipitation

Total protein of cells was extracted by radioimmunoprecipitation (RIPA) assay, and protein concentrations were measured using BCA Protein Assay Kit (Biomed Biology, Beijing, China). Cell lysates (10 μg) were separated by 10%‐15% SDS‐PAGE gels, transferred onto a PVDF membrane (Millipore, MA, USA) and blotted with monoclonal antibodies for Atg7, Atg5, p62, LC3, His, β‐actin (Cell signal Technology, MA, USA) and anti‐ASPP2 (Clone DX54.10) (Sigma‐Aldrich, MO, USA). Specific proteins were detected with enhanced chemiluminescence (Pierce SuperSignal, Thermo Fisher Scientific Inc, Rockford, IL, USA).

Cell lysates (500 μg of protein) were incubated with 2 μg of His antibody for 3 hours at 4°C and then with protein A/G agarose (Santa Cruz, Texas, USA) for 2 hours with gentle shaking. Beads were collected by centrifugation, washed 3 times with RIPA buffer and eluted by incubation with SDS loading buffer at 95°C for 5 minutes. Immunoblot assay was performed with anti‐HSF1 monoclonal antibody.

### 2.4 Quantitative real‐time PCR analysis

Total RNA was isolated from cells 12 hours post‐transfection using RNeasy Mini Kit (Qiagen, Hilden, Germany). Reverse‐transcription was performed with SuperScript III First‐Strand Synthesis System (Invitrogen, CA, USA). RT‐qPCR was performed on ViiA 7DX RT‐PCR system with Fast SYBR Green Master Mix (ABI, MA, USA). Relative transcript levels of target genes were normalized with GAPDH mRNA levels. Specific primers used for RT‐qPCR in this study were listed in Table 2.

**TABLE 2:** Primers used by real‐time PCR

	Forwards primers (5’‐3’)	Reverse primers (5’‐3’)
Atg7	AAGAAATAATGGCGGCAGCT	ACCCAACATCCAAGGCACTA
Atg5	AGAGTAAGTTATTTGACGTT	TCATAACCTTCTGAAAGTGCT
LC3	ATGCCGTCGGAGAAGACCTT	TGCTGCTCTCGAATAAGTCG
P62	GCCACAGCCTCGGTCAAGAT	TGAGTGAAGGACGGGTCGAG
GAPDH	GAGCCACATCGCTCAGACAC	GGTGCAGGAGGCATTGCTGA

### 2.5 Immunofluorescent staining

Cells were washed with cold PBS twice, fixed with 4% paraformaldehyde/PBS for 10 minutes, incubated with 1% Triton X‐100 in PBS for 5 minutes and blocked with 2% BSA and 1% goat serum/PBS for 1 hours, followed by incubation with appropriately diluted primary antibodies for 1 hours at room temperature. Then, the cells were incubated with Alexa Fluor 488‐ or 594‐conjugated secondary antibody (Sigma‐Aldrich, MO, USA). DAPI (4,6‐diamidino‐2‐phenylindole) was used to stain the nuclei. Figures were generated and analysed with Adobe Photoshop (Adobe Systems Inc, version 7.0, San Jose, CA).

### 2.6 Chromatin immunoprecipitation assay

ChIP assay and ChIP‐PCR were performed to determine the influence of ASPP2 on the capacity of HSF1 binding to Atg7 promoter. 6 × 10^6^ cells were cross‐linked with 1% formaldehyde for 10 minutes at room temperature, and the reaction was stopped by adding glycine to a final concentration of 0.125 mol/L. Cells were washed with cold PBS and re‐suspended in cold Lysis RIPA buffer (150 mmol/L NaCl, 1% NP‐40, 0.5% deoxycholate, 0.1% SDS, 50 mmol/L Tris (pH 8.0), 5 mmol/L EDTA) containing protease inhibitors including 1 mmol/L PMSF (phenylmethylsulfonyl fluoride), leupeptin (10 μg/ml) and pepstatin (10 μg/mL) ). Cell lysates were sonicated to yield chromatin fragments of approximately 600‐1000 bp. Protein extract was pre‐cleared with 50 μL of Pro A/DNA slurry (25 μL/25 μL) for 30 minutes at 4°C. After centrifugation, supernatants were used for IP. A total of 1 μg of HSF1 antibody (Cell Signal Technology, MA, USA) were added to pre‐clear supernatant, and immunoprecipitation was performed by rocking overnight at 4°C. Approximately 50 μL of Protein A/G beads were added, and incubation was conducted for another 2 hours at 4°C. Immunocomplexes were washed twice with RIPA buffer, four times with IP wash buffer (100 mmol/L Tris [pH 8.0], 500 mmol/L LiCl, 1% NP‐40, 1% deoxycholic acid) and twice more with RIPA buffer. Then, 2 μL 5 mg/ml RNase A and 12 μL 5 mol/L NaCl (bring to 0.2 mol/L) were added for incubating at 65°C for at least 6 hours. DNA was purified with Qiagen PCR spin columns. Purified DNA was analysed by PCR using the following primers: HSF1 forward: 5'‐GTCAGCAAAGGGTGGTGGGATTATC‐3'; HSF1 reverse: 5'‐GTCAGCAAAGGGTGGTGGGATTATC‐3'.

## 3 RESULTS

### 3.1 HBV‐induced hepatocyte autophagy in a viral dose‐dependent manner

**FIGURE 1:**
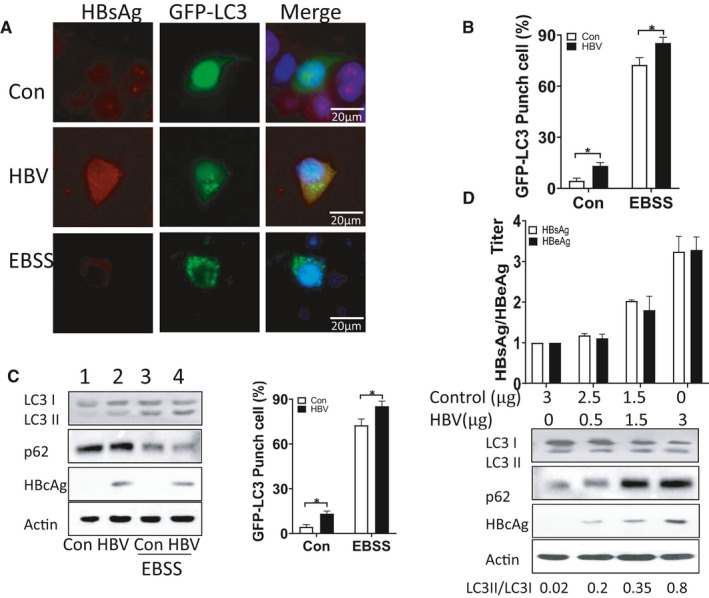
A, Immunofluorescence images of HepG2 cells transfected by HBV and GFP‐LC3 plasmid. Cells were stained with anti‐HBc antibody (red) 48 h after plasmid transfection. B, Percentage of cells with GFP‐LC3 puncta. Bars were cells from four groups, including cells transfected with control and HBV plasmid and cells transfected with control and HBV plasmid followed by starvation, respectively. Asterisks indicate the statistical significance (*P* < .05). C, Immunoblot analysis of four cell groups with different treatments, respectively. Bar graph of LC3‐II/LC3‐I ratio from four groups with HepG2 cells. D, HBsAg/HBeAg titre and immunoblot analysis of HepG2 cells transfected with different amounts of HBV plasmids. Data represent mean (±SD) from 3 independent experiments. *P < .05; **P < .01

HBV is reported to induce autophagy and can be simultaneously profited from autophagy for self‐replication.^6^ In the present study, HBV infection could induce hepatocyte autophagy, especially in nutrient‐deficient environment. HepG2 cells were co‐transfected with HBV plasmid (Gift from Dr James Ou, University of South California), control plasmid and GFP‐LC3 plasmid. Cells treated with 12‐hour starvation were used as the positive control. As shown in Figure 1A, GFP‐LC3 puncta were observed in cells treated with starvation and HBsAg positive cells as well. By calculating the population of GFP‐LC3‐positive cells, HBV‐transfected cells had more GFP‐LC3 puncta (about 18%) when compared with the cells transfected with control plasmid (about 5%) (*P* < .05) (Figure 1B). In addition, Earle's Balanced Salt Solution (EBSS) treatment was used as the mimic starvation condition. The cells transfected with HBV and control plasmid were then treated with EBSS for 12 hours. After EBSS treatment, GFP‐LC3 puncta revealed a dramatic increase in HBV‐transfected cells when compared with control plasmid‐transfected cells (Figure 1B column 3 and 4), with a statistically significant difference (*P* < .01). The similar trend of LC3 protein expression is also detected by using immunoblotting (Figure 1C). As shown in Figure 1C, HBV infection increased LC3‐II/LC3‐I ratio to 0.25 (*P* < .05); and the LC3‐II/LC3‐I ratio in EBSS‐treated cells was approximately 0.6 (Figure 1C, column 3). Compared with the cells subjected to nutrient‐starvation treatment alone, HBV‐infected cells combined with nutrient‐starvation treatment exhibited the highest LC3‐II/LC3‐I ratio up to 1.6 (Figure 1C right part, column4, *P* < .01). All these data revealed that HBV infection could induce autophagy, especially in nutrient‐starvation conditions. To further identify the effect of HBV infection severity on autophagy, HepG2 cells seeded in 6‐well culture plates were transfected with different amounts of HBV plasmid (0, 0.5, 1.5 and 3 μg/per well). After 48‐h transfection, the supernatant of cell culture was collected to determine the release of HBV antigens by enzyme‐linked immunosorbent assay (ELISA). HBsAg and HBeAg present concomitant increase with the increased amounts of HBV plasmid. To be noticed, the increase in LC3‐II/LC3‐I ratio exhibited the similar trend as the secretion level of HBV antigens and the expression of HBV core protein (HBc) (Figure 1D). These results indicate that HBV can induce hepatocyte autophagy in a virus dose‐dependent manner.

### 3.2 ASPP2 inhibited HBV replication by altering HBV‐induced autophagy in hepatocytes

**FIGURE 2:**
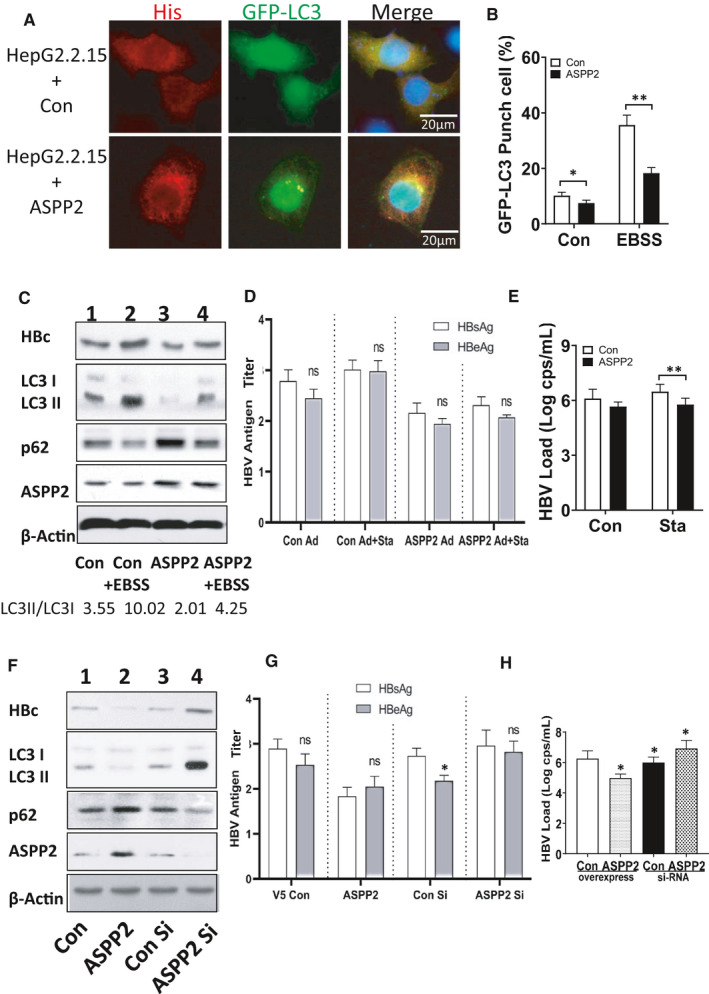
HepG2.2.15 cells were co‐transfected with His‐ASPP2/His‐control plasmid and GFP‐LC3 plasmid, then treated with EBSS treatment for 12 h. A, Immunofluorescence images of GFP‐LC3 puncta. Cells were stained with anti‐His antibody (red) 12 h after EBSS treatment. B, Percentage of cells with GFP‐LC3 puncta. C, Immunoblot analysis of four cell groups upon different treatments, respectively. D, HBsAg/HBeAg titre of four cell groups was detected by ELISA. E, HBV viral load was analysed by real‐time PCR from four cell groups. HepG2.2.15 cells were also treated with ASPP2 siRNA or control siRNA transfection. F, Immunoblot analysis for the expression of HBc and LC3 among His‐control plasmid, His‐ASPP2, control siRNA and ASPP2 siRNA‐transfected cells. G, HBsAg/HBeAg titre of four cell groups with different treatments. H, HBV viral load was analysed by real‐time PCR. Data represent mean (±SD) from 3 independent experiments. *P ˂ .05; **P ˂ .01; ***P ˂ .001

In our previous study, ASPP2 could inhibit autophagy induced by starvation and other autophagy inducers in multiple liver diseases.^14^, ^19^ The effect of ASPP2 on HBV‐induced autophagy remains unclarified. HepG2.2.15 cells, HBV genome‐integrated cell lines, were co‐transfected with ASPP2 plasmid and GFP‐LC3 plasmid, followed by EBSS treatment. Analysed by Immunofluorescent staining, ASPP2‐overexpressed HepG2.2.15 cells had less GFP‐LC3 puncta when compared with the control cells (Figure 2A,B). After HBSS treatment, the level of cell autophagy increased, and ASPP2 overexpression significantly reduced autophagy (Figure S1). It further showed that ASPP2 could significantly reduce hepatocyte autophagy caused by starvation and HBV infection. ASPP2 overexpression could decrease the percentage of GFP‐LC3 puncta cells (*P* < .05), especially in EBSS‐treated HepG2.2.15 cells (*P* < .01). We next investigated the function of ASPP2 on HBV‐related protein expression, HBV replication and HBV antigen release. Evaluating by Western blotting, the overexpression of ASPP2 in HepG2.2.15 cells decreased LC3‐II expression and simultaneously reduced HBc expression (Figure 2C column 3 compared with column 1, column 4 compared with column 2). We collected the supernatant to detect HBV antigen release by ELISA. The release of HBsAg and HBeAg was reduced in supernatant of ASPP2‐overexpressed cells consistently (Figure 2D). HBV viral load was reduced in ASPP2‐overexpressed HepG2.2.15 cells to approximately 1 log (Figure 2E).

In this study, siRNA was also used to inhibit ASPP2 expression in HepG2.2.15 cells. In order to control the expression efficiency of ASPP2 and ASPP2‐specific siRNA, these proteins and corresponding targets were examined through Western blot (Figure S2). The increase in ASPP2 signal was observed in His‐ASPP2‐transfected cells, but a decrease in ASPP2 siRNA‐transfected cells when compared with the controls (Figure S2). ASPP2 siRNA 2 was chosen to construct ASPP2 knockdown model. The decrease of LC3‐II in His‐ASPP2‐transfected cells and the increase in ASPP2 siRNA‐transfected cells were observed (Figure 2F). Similarly, the reduced HBsAg and HBeAg in ASPP2‐overexpressed cell supernatant were also determined, but the increase in ASPP2 siRNA‐transfected cell supernatant was observed (Figure 2G). HBV viral load increased the expression or release of HBsAg and HBeAg in ASPP2 siRNA‐transfected cells when compared to control siRNA‐transfected cells (Figure 2H). These results confirmed that ASPP2 could inhibit HBV replication through altering HBV‐induced hepatocyte autophagy.

### 3.3 ASPP2 attenuated HBV‐induced hepatocyte autophagy in a p53‐independent manner

**FIGURE 3:**
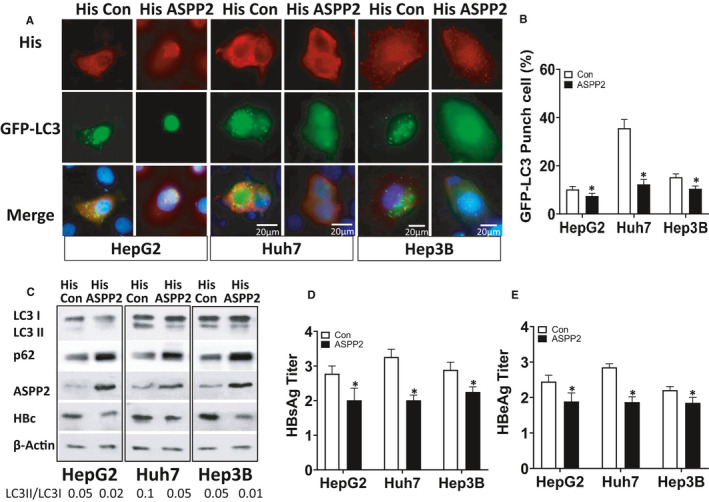
HepG2, Huh7 and Hep3B cells with intrinsic different p53 status were co‐transfected with His‐control/His‐ASPP2 plasmid, GFP‐LC3 plasmid and HBV plasmid. A, Immunofluorescence images of GFP‐LC3 puncta. Cells were stained with anti‐His antibody (red) 48 h after transfection. B, Percentage of cells with GFP‐LC3 puncta. C, Immunoblot analysis of three cell lines upon treatments. D, HBsAg titre of three cell lines upon treatments. E, HBeAg titre of three cell lines upon treatments. Data represent mean (±SD) from 3 independent experiments

As ASPP2 had a close relationship with p53 and p53 mutation or dysfunction in many liver diseases, it is essential for us to examine whether the role of ASPP2 in autophagy is dependent on the presence of p53. Three hepatoma cell lines (Huh7, HepG2 and Hep3B) with intrinsic different p53 status were used to evaluate the function of ASPP2 on HBV‐induced autophagy. Three cell lines were co‐transfected with GFP‐LC3 and HBV plasmid, and then treated with EBSS. Through immunofluorescent staining, ASPP2‐transfected cells had less GFP‐LC3 puncta than His‐control‐transfected cells. The similar phenomena were observed in three cell lines (Figure 3A). By calculating the percentage of GFP‐LC3 puncta cells, ASPP2‐transfected cells had lower percentage of GFP‐LC3 puncta cells (Figure 3B). Cell lysates were analysed by WB. After His‐ASPP2 transfected, the LC3‐II/LC3‐I ratio was decreased to 0.02, 0.05 and 0.01 in HepG2, Huh7 and Hep3B cells, respectively (Figure 3C). These results demonstrated that ASPP2 overexpression could inhibit autophagy regardless of p53 status. HBV C protein (HBc) had a low expression level in ASPP2‐overexpressed cells, suggesting that ASPP2 could inhibit HBV replication. The culture supernatant was collected to compare the antigen‐releasing levels. The titres of HBsAg and HBeAg in cell culture supernatant revealed a declining trend in ASPP2‐overexpressed cells (Figure 3D). All these data showed that ASPP2 could attenuate hepatocyte autophagy and inhibit HBV replication in a p53‐independent manner.

### 3.4 ASPP2 inhibited HBV replication through attenuating Atg7 expression

**FIGURE 4:**
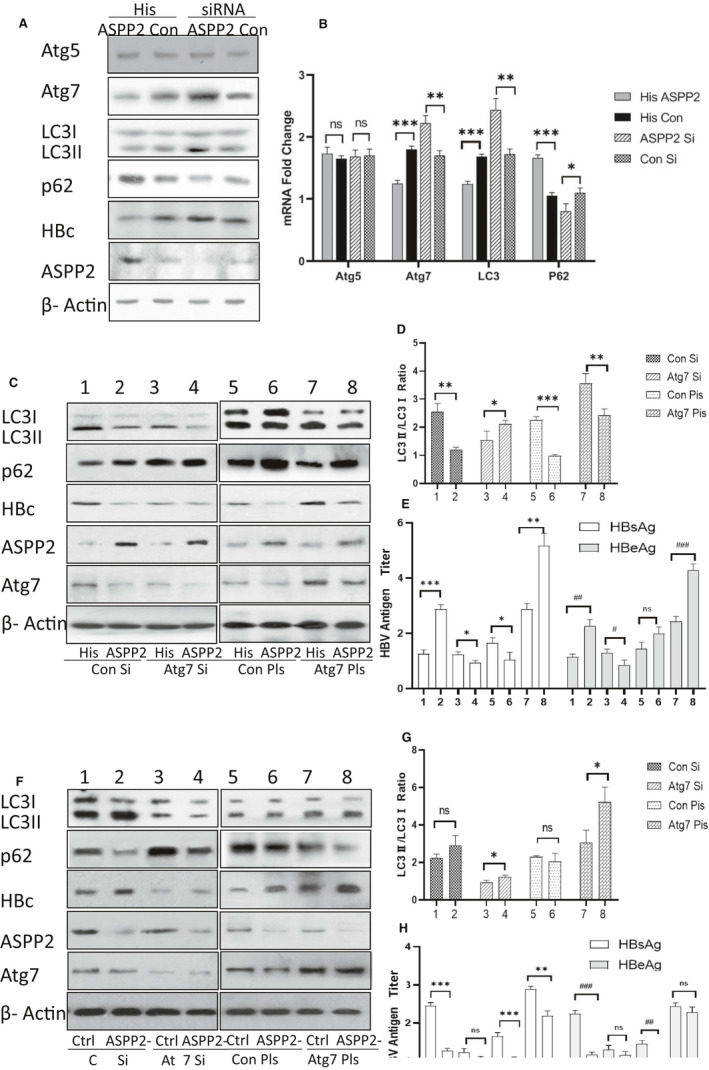
HepG2.2.15 cells were transfected with His‐control plasmid, His‐ASPP2, control siRNA and ASPP2 siRNA, respectively. A, Immunoblot analysis of autophagy‐related proteins from four cell groups. B, The relative transcript levels of Atg5, Atg7, LC3 and p62 from four cell groups analysed by using real‐time PCR. HepG2.2.15 cells were co‐transfected with His‐ASPP2/His‐control plasmid and control siRNA/Atg7 siRNA or control plasmid/Atg7 plasmid. Post‐transfection for 48 h, cells and cell culture supernatant were collected to analyse protein expression and HBV antigen release. C, Immunoblot analysis for protein expression in eight cell groups with different treatments. D, Bar graph of LC3‐II/LC3‐I ratio from eight HepG2.2.15 cell groups. E, HBsAg/HBeAg titre of eight cell groups detected by ELISA. HepG2.2.15 cells were co‐transfected with control siRNA/ASPP2 siRNA and control siRNA/Atg7 siRNA or control plasmid/Atg7 plasmid. Post‐transfection for 48 h, cells and cell culture supernatant were collected to analyse protein expression and HBV antigen release. F, Immunoblot analysis for protein expression in eight cell groups with different treatments. G, Bar graph of LC3‐II/LC3‐I ratio from eight HepG2.2.15 cell groups. H, HBsAg/HBeAg titre of eight cell groups detected by ELISA. Data represent mean (±SD) from 3 independent experiments. * and ^#^, *P* ˂ .05; ** and ^##^, *P* ˂ .01; *** and ^###^, *P* ˂ .001

HBV can initiate autophagy at the early stage.^6^ In order to explore the regulatory mechanism of ASPP2‐associated autophagy, the expression of autophagy‐related proteins was examined. HepG2.2.15 cells were transfected with His‐ASPP2 or ASPP2 siRNA to up‐ or down‐regulate ASPP2 expression. According to the analysis by Western blotting, ASPP2 overexpression could attenuate LC3‐II expression, but stimulate p62 expression (Figure 4A), indicating that ASPP2 could inhibit autophagy. Importantly, Atg5 remained at the same level between both groups, but a very low expression level of Atg7 in His‐ASPP2‐transfected cells was detected. A similar trend in mRNA expression levels for these autophagy‐related molecules (Figure 4B) was observed through RT‐PCR analysis. Furthermore, ASPP2 siRNA was used to silence ASPP2 in HepG2.2.15 cells. As expected, LC3‐II/LC3‐I ratio revealed an obvious increase and p62 exhibited a significant decrease in ASPP2‐silent cells. The expression of Atg7 and its mRNA present an evident increase in these cells than the control siRNA‐transfected cells. Atg7, as the homology to E1 ubiquitin activation enzymes, plays a critical role in LC3 lipidation. Therefore, the expression level of Atg7 may be one of the major reasons for inducing the change of LC3‐II. In order to determine the role of Atg7 in ASPP2‐mediated regulation of HBV replication, a plasmid carrying Atg7 gene or Atg7 siRNA was used to up‐regulate or down‐regulate Atg7 expression in HepG2.2.15 cells. Atg7 expression was decreased in cells transfected with His‐ASPP2 plasmid (Figure 4C column 2), as well as LC3‐II/LC3‐I ratio and HBc expression. As shown in Figure 4C column 4, cells co‐transfected with His‐ASPP2 and Atg7 siRNA plasmids had the lowest expression level of Atg7 and LC3‐II/LC3‐I ratio (Figure 4C,D). The HBsAg/HBeAg releasing level in supernatant detected by ELISA also present as the lowest level when compared with other treatments (Figure 4E). Conversely, the cells co‐transfected with Atg7 plasmid and ASPP2 siRNA had the highest expression level for Atg7 and LC3‐II/LC3‐I ratio (Figure 4F column 8 and Figure 4F column 8, Figure 4G), and the release of HBsAg/HBeAg in supernatant was also the highest (Figure 4H). These results indicated that ASPP2 could inhibit HBV replication through attenuating Atg7 expression.

### 3.5 ASPP2 bound to HSF1 in cytoplasm and inhibited HSF1‐mediated Atg7 transcription

**FIGURE 5:**
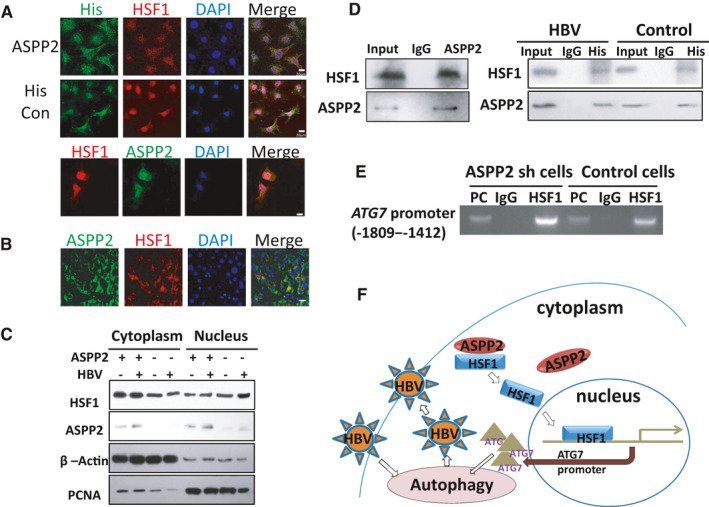
A, HepG2.2.15 cells were co‐transfected with His‐ASPP2 and HSF1 plasmid. Subcellular localization of exogenous HSF1 and ASPP2 was detected by immunostaining with anti‐HSF1 (red) and anti‐His (green) antibodies. Endogenous ASPP2 and HSF1 were detected by immunostaining with anti‐HSF1(red) and anti‐ASPP2 (green) antibodies. B, Immunofluorescent staining of liver section from normal liver tissues. C, His‐ASPP2 and HBV plasmids were co‐transfected into Huh7 cells. Post‐transfection for 48 h, proteins in cytoplasm and nucleus were isolated, and HSF1 and ASPP2 localization were analysed by immunoblot analysis. D, The binding of endogenous ASPP2 and HSF1 in HepG2.2.15 cells were analysed by co‐immunoprecipitation with anti‐ASPP2 antibody, followed by probing with anti‐HSF1 antibody (left). Total proteins from Huh7 cells were performed for co‐immunoprecipitation with His‐tag antibody, followed by probing with anti‐HSF1 antibody (Right). E, ChIP assay of Atg7 promoter containing HSF1‐binding sites from −1809 to −1412 was performed to measure the effect of ASPP2 on the binding activity of HSF1 when Huh7 cells were transfected with ASPP2 siRNA. F, A model for underlying mechanisms of ASPP2 to modulate autophagy. In normal circumstances, ASPP2 and HSF1 were co‐located in cytoplasm. When cells were infected with HBV, autophagy was triggered. HSF1 was increased and translocated into nucleus to bind to Atg7 promoter and up‐regulate Atg7 expression, thus leading to further induced autophagy. The induced autophagy can promote HBV replication. Overexpressed ASPP2 can bind to HSF1 in cytoplasm and constrain its nuclear re‐localization, thus down‐regulating Atg7 expression and leading to autophagy inhibition. Therefore, the suppressed autophagy can reduce HBV replication

ASPP2 overexpression inhibited HBV replication by reducing both mRNA and protein levels of Atg7, so it is necessary to identify how ASPP2 down‐regulates Atg7 transcription. HSF1 was reported to be a factor that could bind to Atg7 promoter under starvation. So, the interaction of ASPP2 and HSF1 in cell models under HBV infection condition was also evaluated. His‐ASPP2 or His‐control plasmid and HSF1 plasmid were co‐transfected into HepG2.2.15 cells. Exogenous His‐ASPP2 was mainly distributed in the cytoplasm as confirmed by immunostaining (Figure 5A), but exogenous HSF1 was located in both cytoplasm and nucleus. When HSF1 was in the cytoplasm, it was partially co‐located with ASPP2 (Figure 5A upper four pictures). In the control plasmid‐transfected cells, HSF1 signal was mainly located in nucleus (Figure 5A middle four pictures). We also investigated the expression of endogenous ASPP2 and HSF1 in HepG2.2.15 cells and found that ASPP2 and HSF1 can partially co‐localize in the cytoplasm (Figure 5A lower four pictures). Immunofluorescence staining of normal liver tissue from a liver transplantation donor showed that both ASPP2 and HSF1 were expressed in the cytoplasm (Figure 5B), and ASPP2 and HSF1 were partially co‐located. Next, we co‐transfected His‐ASPP2 and HBV plasmids into Huh7 cells to investigate the intracellular localization of HSF1 under ASPP2 overexpression and HBV infection condition. Immunoblotting showed that total HSF1 expression revealed an obvious increase in HBV‐infected Huh7 cells (Figure 5C) and large amount HSF1 entered into nucleus (Figure 5C column 4 and 8). However, only small amount HSF1 entered into nucleus in HBV‐infected Huh7 cells with ASPP2 overexpression (Figure 5C column 2 and 6). From these results, we suggest that ASPP2 can bind to HSF1 and prevent its entrance into nucleus. We further verified the association of ASPP2 and HSF1 in cytoplasm by co‐IP. HepG2.2.12 cells were used here to detect the endogenous binding of ASPP2 and HSF1. Huh7 cells which transfected HBV plasmid and control plasmid were used here to analyse the binding of ASPP2 and HSF1 under HBV infection. As expected, the interaction of ASPP2 and HSF1 was confirmed (Figure 5D). As ASPP2 can bind to HSF1 in the cytoplasm and prevent HSF1 nucleus translocation, whether their binding could affect the transcription of Atg7 is highly required to be confirmed. The transcriptional activation of Atg7 by HSF1 in the context of ASPP2 silence was confirmed by using chromosome immunoprecipitation technique. The region of Atg7 promoter from −1809 and −1412 was identified to bind HSF1 (Figure 5E).^21^ ChIP assay showed that HSF1 could bind to Atg7 promoter and suppress ASPP2, thereby enhancing the binding of HSF1 to Atg7 promoter.

We also found that when HSF1 and ASPP2 were co‐transfected into Huh7 cells, the decrease in Atg7 transcription caused by ASPP2 overexpression was offset (Figure S3). Taking together, ASPP2 can interact with HSF1 to prevent the activation of Atg7, thus leading to autophagy inhibition in HBV‐infected cells.

## 4 DISCUSSION

Autophagy uses endocytosis to digest and reuse the damaged organelles for achieving a stable internal environment for cell survival. The role of autophagy in virus infection is complicated. Some viruses, such as HBV and HCV, can induce autophagy, but autophagy also can promote the replication of these viruses, which is not beneficial for the elimination of viruses by the host.^22^ S protein of HBV (HBS) can induce autophagosome formation by triggering endoplasmic reticulum (ER) stress and initiating unfolded protein response (UPR).^5^ X protein of HBV (HBX) can activate MAPK (mitogen‐activated protein kinase), ROS/JNK (reactive oxygen species/c‐Jun NH2‐terminal kinase) and Sirt1 signal pathways.^23^, ^24^, ^25^ HBx can also interact with V‐ATPase to inhibit lysosomal acidification, thus impairing lysosomal degradation.^26^ Recently, HBV is reported to promote itself maturation through subverting autophagy elongation complex Atg5‐12/16L1.^27^ The inhibition of autophagy can reduce HBV replication. For example, in vitro, the suppression of PI3KC3 or inhibition of Atg7 can result in the reduction in HBV DNA.^6^ Some drugs also can promote HBV replication by induced autophagy. Cisplatin has been confirmed to promote HBV replication and autophagy through ROS/JNK and AKT/mTOR signalling.^28^ It is reported that miR‐146a‐5p can mediate a positive feedback loop by regulating autophagy‐induced HBV replication via targeting the XIAP (X‐linked inhibitor of apoptosis protein)‐mediated MDM2 (murine double minute 2)/p53 axis.^29^ Therefore, logically, autophagy inhibition is expected to be a prospective anti‐HBV pathway.

Our data have demonstrated that ASPP2 can inhibit autophagy, thus reducing HBV replication. HBV infection can trigger HSF1 nucleus translocation and ASPP2 can bind to HSF1 in cytoplasm, thus limiting the nuclear translocation of HSF1, and reducing the transcriptional activation of Atg7. The inhibition of Atg7 expression in turn can lead to a decrease in HBV replication (Figure 5E).

ASPP2 is a member of p53‐binding protein family that can bind to p53 and activate p53‐mediated apoptotic pathway.^30^ Recent studies have documented that ASPP2 is involved in a number of cellular signal pathways, many of which are not dependent on the presence of p53.^31^ HBV infection induces a series of liver‐related pathological reactions to result in chronic hepatitis, cirrhosis and even hepatocellular carcinoma. The overexpression of ASPP2 is reported to play an inhibitory role in the development of HCC. Recombinant human adenovirus ASPP2 is also found to play a synergistic inhibitory effect on hepatocellular carcinoma formation by influencing protein expression associated with proliferation, apoptosis, autophagy and vascular growth via a p53‐independent pathway in vivo.^32^ In a p53‐independent manner, ASPP2 can enhance chemotherapeutic sensitivity through down‐regulating XIAP expression in hepatocellular carcinoma.^33^

The known effects of ASPP2 on apoptosis and tumour suppression may play beneficial roles in the progression of different HBV‐infected liver diseases. Taking into consideration the function of ASPP2 on the suppression of HBV replication by reducing hepatocyte autophagy, ASPP2 is expected to be an ideal treatment strategy for HBV infection‐related liver diseases.

In addition, we have noted that ASPP2 can inhibit autophagy in the absence of p53. All these results suggest that ASPP2 has a multi‐faceted beneficial effect as a therapeutic target, thereby contributing to the overall treatment efficiency of chronic hepatitis B.

In summary, ASPP2 is probably a meaningful target of preventing and controlling HBV. Further mechanistic studies should be explored in vitro and in vivo.

## CONFLICT OF INTEREST

No potential conflict of interest was reported by the authors.

## AUTHOR CONTRIBUTIONS

Shanshan Wang: Data curation (equal); Formal analysis (equal); Resources (equal); Writing‐original draft (equal). Yu Sun: Formal analysis (equal); Funding acquisition (equal); Writing‐original draft (equal). Yang Wang: Data curation (equal); Formal analysis (equal). Anna Wang: Formal analysis (equal); Writing‐original draft (equal). Buxin Kou: Formal analysis (equal). Yang Che: Formal analysis (equal). Dexi Chen: Funding acquisition (equal); Writing‐review & editing (equal). Yulin Zhang: Writing‐review & editing (equal). Ying Shi: Conceptualization (equal); Data curation (equal); Formal analysis (equal); Funding acquisition (equal); Investigation (equal); Methodology (equal); Software (equal); Validation (equal); Writing‐original draft (equal); Writing‐review & editing (equal).

## Supporting information

Fig S1

Fig S2

Fig S3
