# Supplementary figures and images for: ASPP2 inhibits hepatitis B virus replication by preventing nucleus translocation of HSF1 and attenuating the transactivation of ATG7
Source: J Cell Mol Med. 2021 Jun 4;25(14):6899–908. doi: 10.1111/jcmm.16699 (PMC8278078; doi:10.1111/jcmm.16699)

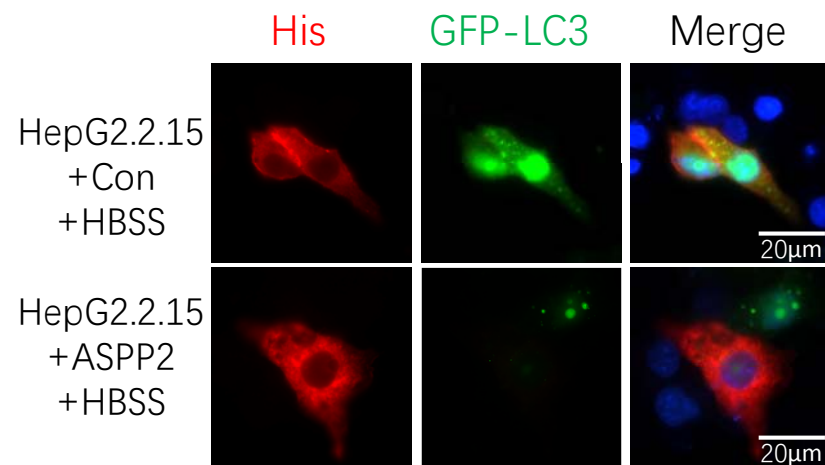

Supplement: Supplementary file 1 — Fig S1 [file JCMM-25-6899-s001.pdf]

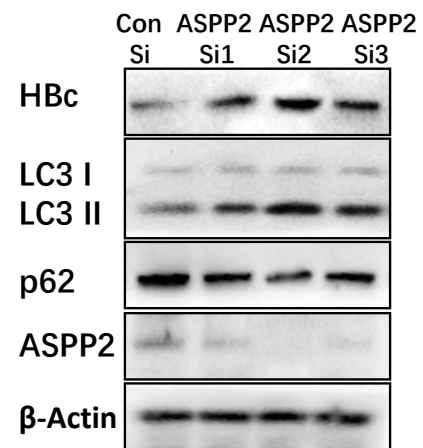

Supplement: Supplementary file 2 — Fig S2 [file JCMM-25-6899-s002.pdf]

**A**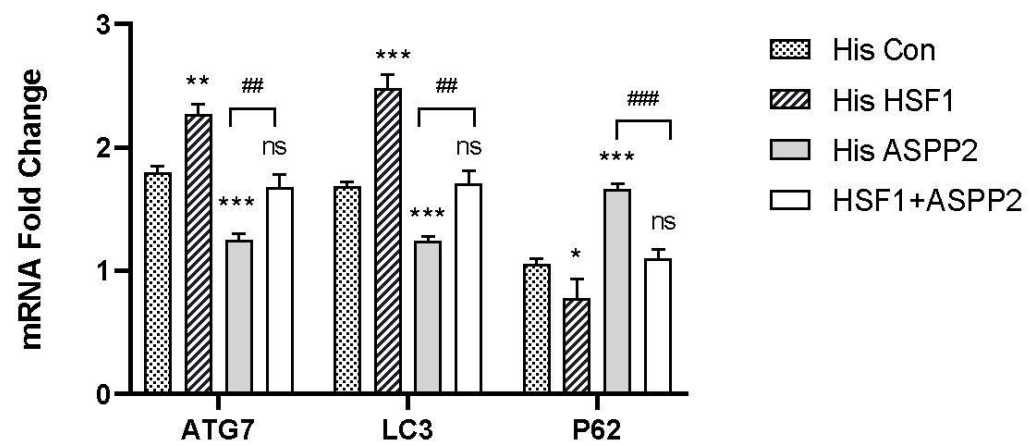**B**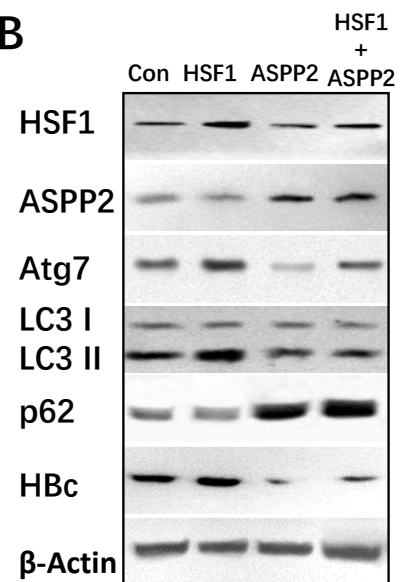

Supplement: Supplementary file 3 — Fig S3 [file JCMM-25-6899-s003.pdf]
